# Phytochemical analysis, antioxidant and metal chelating capacity of *Tetrapleura tetraptera*

**DOI:** 10.1016/j.heliyon.2019.e02762

**Published:** 2019-11-14

**Authors:** Stephen Adusei, John K. Otchere, Prince Oteng, Richard Q. Mensah, Emmanuel Tei-Mensah

**Affiliations:** a Department of Environmental Science, University of Cape Coast, Cape Coast, Ghana; b Department of Laboratory Technology, University of Cape Coast, Cape Coast, Ghana; c Department of Chemistry, University of Cape Coast, Cape Coast, Ghana; d Department of Biomedical Engineering, University of Ghana, Legon, Ghana; e Department of Food and Postharvest Technology, Koforidua Technical University, Koforidua, Ghana

**Keywords:** Food science, Pharmaceutical chemistry, *Tetrapleura tetraptera*, Phytochemicals, Antioxidant, Metal chelating capacity

## Abstract

*Tetrapleura tetraptera,* claim to be beneficial for curing of human ailments. In this study, we determined phytochemicals, antioxidant and metal chelating capacity in the pulp, seeds and whole fruit (mixture of pulp and seeds) of *T. tetraptera*. Phytochemicals (flavonoids, alkaloids, terpenoids, tannins, steroids, saponins and phenols) were examined in aqueous and ethanolic extracts of the pulp, seeds and whole fruit. The recovery of all compounds was satisfactory, in the range of 90%–108%. The screening test revealed the presence of many phytochemicals in either one or both extracts. After the qualitative analysis, UV-Spectrophotometer was used to quantify phenols, flavonoids, saponins and alkaloids where higher phytochemical concentrations were recorded in the pulp followed by whole fruit and then the seeds. The metal chelating capacity was determined using EDTA standard, and was in the order of pulp>whole fruit>seeds. Also, the pulp was established to exhibit much antioxidant activity as compared to the whole fruit or seeds. This study therefore supports the use of *T. tetraptera* in traditional systems of medicine owing to its active chemical compounds, and has given many vital insights on which part of *T. tetraptera* fruit to consume as concentrations of these compounds varies in the pulp, seeds and whole fruit.

## 1 Introduction

*Tetrapleura tetraptera* is a flowering plant in the pea family native to Western Africa. It belongs to the order *fabales* and family *fabaceae*. It is a deciduous tree commonly known as Aridan tree (Uyoh et al., 2013). It is mostly found in Ghana where it is known in the local dialect as “prekese”, which is applied in soup preparation as a result of its nice fragrance and suspected medicinal capabilities. In Ghana, the fruit, seeds and flowers are used in countless ways including perfumes, pomades preparation from palm oil, production of some alcoholic beverages and as flavouring for biscuits (Aladesanmi, 2007). Also, in West Africa, *Tetrapleura tetraptera* is mainly used as a spice, medicine and as dietary supplement rich in vitamins (Osei-Tutu et al., 2010). The extract of this plant is known for its anti-inflammatory properties and this advocates its inhibitory impacts against certain human pathogens. According to Ozaslan et al. (2016) the plant is mostly used in the management of convulsion, leprosy, inflammation and rheumatic pains, schistosomiasis, asthma, hypertension and also recommended for fast relief of ailment such as malaria fever. The medicinal value of this plant lies in the bioactive phytochemical constituents that produce certain physiological action on the human body (Akinmoladun et al., 2007).

Medicinal plants contain some organic compounds which provide definite physiological action on the human body and these bioactive substances includes tannins, alkaloids, carbohydrates, triterpenoids, steroids and flavonoids (Edogo et al., 2005). These organic compounds are known as phytochemicals. Phytochemicals (from the Greek word phyto, meaning plant) are biologically active, naturally occurring chemical compounds found in plants, which provide health benefits for humans further than those attributed to macronutrients and micronutrients (Jasiem, 2016). They protect plants from diseases, damage and contribute to the plant's colour, aroma and flavour. In general, the plant chemicals that protect plant cells from environmental hazards such as pollution, stress, drought, ultraviolet (UV) exposure and pathogenic attach are referred to as phytochemicals (Koche et al., 2016).

Chelation is bonding of molecules to metal ions. Chelating agents are organic or inorganic compounds that have the ability to bind to toxic metal ions to form complex structures which are easily excreted from the body, removing them from intracellular or extracellular spaces (Swaran and Pachauri, 2010). Antioxidants are substances that neutralize the harmful free radicals in our bodies. Antioxidants act as “free radical scavengers” and hence prevent or slow the damage done by these free radicals. Antioxidants function as reducing agents, ultimately eliminating free radical intermediates and inhibiting further oxidation (Phang et al., 2013). Many reports have revealed the phytochemical constituents of *T. tetraptera* and demonstrated its antioxidant activity and metal chelating capacity, mostly in the pulp of the plant (Uyoh et al., 2013). In this recent study, we identified and quantified phytochemicals in aqueous and ethanolic extracts, determined the antioxidant activity as well as the metal chelating capacity of the pulp, seeds and whole fruit (pulp and seeds mixture) of *T. tetraptera*.

## 2 Materials and methods

### 2.1 Sample collection

Pods of *T. tetraptera* (50 samples) were collected in February 8th, 2018 from the science market in the University of Cape Coast, Ghana and were botanically identified and confirmed by a plant taxonomist at the Department of Agriculture, University of Cape Coast. The samples were kept in a polyethylene bag and transported to the laboratory until processing for analysis.

### 2.2 Sample preparation

The dried pods of *T. tetraptera* were washed gently with tap water and then with distilled water, air-dried and crushed to remove the seeds from the pods. The seed-free crushed pods (pulp), seeds as well as the whole fruit were pulverized separately into coarse powder with mortar and a pestle. The powdered samples were kept distinctly in airtight containers until required for further work.

### 2.3 Sample extraction

The aqueous and ethanolic extracts of pulp, seeds and whole fruit of *T. tetraptera* were prepared using cold maceration method of extraction as described by Ncube et al. (2008). Exactly 50 g each of the coarsely powdered pulp, seeds and whole fruit were kept in contact with 250 ml of the solvents (distilled water and ethanol) in stoppered containers. They were then allowed to stand at room temperature for 72 h with frequent agitation until the soluble matter got dissolved. The mixtures were then strained, the marc (the damp solid material) pressed and the combined liquids clarified by filtration through whatman No.1 filter paper (125 mm) to get filtrate as extracts. The aqueous and ethanolic extracts were concentrated at 100 °C and 78 °C respectively to one-quarter of the original volume using rotary evaporator. The dried extracts were then stored in desiccators until required for use where they were dissolved in appropriate volume of solvents to the desired concentration.

### 2.4 Phytochemical analysis

#### 2.4.1 Qualitative phytochemical tests

The aqueous and ethanolic extracts of the pulp, seeds and whole fruit of *T. tetraptera* were used to screen for the presence of flavonoids, alkaloids, terpenoids, tannins, steroids, saponins and phenols. The screening tests for these major phytoconstituents were carried out using standard qualitative procedures as described by Trease and Evans (2002) and Adegoke et al. (2010).

##### 2.4.1.1 Detection of flavonoids (Alkaline reagent test)

Extracts (0.2 g) were treated with six drops of 2% sodium hydroxide solution. The formation of intense yellow colour, which developed into a colourless solution on addition of dilute acid, gave an indication of the presence of flavonoids in the extracts (Adegoke et al., 2010).

##### 2.4.1.2 Detection of alkaloids (Mayer's test)

Extracts (0.5 g) were dissolved in 5 ml of 1% dilute hydrochloric acid and filtered. Filtrate was treated with Mayer's reagent (Potassium mercuric iodide). The formation of a yellow coloured precipitate gave a positive result for alkaloids in the extracts (Adegoke et al., 2010; Trease and Evans, 2002).

##### 2.4.1.3 Detection of terpenoids (Salkowski's test)

To 0.1 g of the extracts, 0.5 ml of chloroform was added followed by 1 ml of concentrated sulphuric acid. The formation of reddish-brown precipitate gave an indication of the presence of terpenoids in the extracts (Trease and Evans, 2002).

##### 2.4.1.4 Detection of tannins (Ferric chloride test)

A mass of 0.2 g of the extracts were mixed with an equal volume of distilled water in a test tube and three drops of dilute ferric chloride was added. The formation of brownish blue or dark colour gave an indication of the presence of tannins in the extracts (Adegoke et al., 2010).

##### 2.4.1.5 Detection of steroids (Liebermann-Burchard's test)

Extracts (0.5 g) were mixed with 2 ml of chloroform. 2 ml of concentrated sulphuric acid was then added to the mixture in a test tube. The appearance of red colour in the lower chloroform layer gave a positive result for steroids in the extracts (Adegoke et al., 2010).

##### 2.4.1.6 Detection of saponins (Foam test)

To 0.2 g of the extracts, 6 ml of distilled water was added and shaken vigorously in a graduated cylinder for 15 min lengthwise. The formation of bubbles or persistent foam for 10 min gave an indication of the presence of saponins in the extracts (Trease and Evans, 2002).

##### 2.4.1.7 Detection of phenols (Ferric chloride test)

To 0.2 g of the extracts, 2 ml of 5% aqueous ferric chloride was added. The formation of bluish colour gave a positive result for phenols in the extracts (Adegoke et al., 2010).

#### 2.4.2 Quantitative phytochemical determinations

Both the aqueous and ethanolic extracts were used in the quantification of detected phytochemicals. Quantitative assay was carried out for total flavonoids, phenols, saponins and alkaloids.

##### 2.4.2.1 Determination of total flavonoid content

The total flavonoids content of the crude extract was determined by aluminum chloride colorimetric method as described by Piyanete et al. (2009). Quercetin was used as standard and flavonoid content determined as quercetin equivalent. From the standard quercetin solution, the following concentrations (10, 25, 50, 75 and 100 μg/ml) were prepared in methanol. 100 μl of each of the quercetin dilution was mixed with 500 μl of distilled water and then with 100 μl of 5% sodium nitrate and allowed to stand for 6 min. Then 150 μl of 10% aluminum chloride solution was added and allowed to stand for 5 min after which 200 μl solution of 1 M sodium hydroxide was added sequentially. The absorbance of this reaction mixture was measured at 510 nm using single beam UV-VIS spectrophotometer (UV mini-1240). The same procedure was repeated for both the aqueous and the ethanolic extracts of the pulp, seed and whole fruit of *T. tetraptera.* All measurements were performed in triplicate for each analysis. The total flavonoids content was determined from the linear equation of a standard curve prepared with quercetin and expressed as mg/g quercetin equivalent (QE) of dry extract.

##### 2.4.2.2 Determination of total phenol content

The total phenols content of *T. tetraptera* extracts was determined using Folin- Ciocalteu reagent following a slightly modified method of Ainsworth and Gillespie (2007). Gallic acid was used as a reference standard for plotting the calibration curve. A volume of 0.5 ml aliquot of 10, 20, 40, 80 and 100 μg/ml gallic acid solutions were mixed with 2 ml of Folin-Ciocalteu reagent (diluted 1:10 with de-ionized water) and were neutralized with 4 ml of sodium carbonate solution (7.5%, w/v). The reaction mixture was incubated at room temperature for 30 min with intermittent shaking for colour development. The absorbance of the resulting blue colour was measured at 765 nm using single beam UV-VIS spectrophotometer (UV mini-1240). The same procedure was repeated for both the aqueous and the ethanolic extracts of the pulp, seed and whole fruit of *T. tetraptera.* All measurements were performed in triplicate for each analysis. The total phenols content was determined from the linear equation of a standard curve prepared with gallic acid and expressed as mg/g gallic acid equivalent (GAE) of dry extract.

##### 2.4.2.3 Determination of total saponin content

The estimation of total saponins content was determined by the method described by Moja et al. (2003) based on vanillin-sulphuric acid colorimetric reaction with some modifications. Exactly 5000 μL of water was added to 100 μL of diosgenin. 500 μL of vanillin reagent (8 g of vanillin in 100 ml of 99.5% ethanol) was added. 5 ml of 72% sulphuric acid was also added and mixed well. This solution was kept in a water bath at 60 °C for 10 min. After 10 min, it was cooled and the absorbance read at 544 nm and recorded. The same procedure was repeated for both the aqueous and the ethanolic extracts of the pulp, seed and whole fruit of *T. tetraptera.* All measurements were performed in triplicate for each analysis. The total saponins content was determined from the linear equation of a standard curve prepared with diosgenin and expressed as mg/g diosgenin equivalent (DE) of dry extract.

##### 2.4.2.4 Determination of total alkaloid content

The total alkaloids content (TAC) of the crude extracts was determined according to the methodology as described by Adegoke et al. (2010). One gram of crude extract of the pulp, seeds and whole fruit of *T. tetraptera* was weighed into a 100 ml beaker and 50 ml of 10% hydrochloric acid in ethanol was added and covered and allowed to stand for 4 h. This was filtered and the filtrate was concentrated at 78 °C to one-quarter of the original volume using a rotary evaporator. 15 drops of concentrated ammonium hydroxide were then added dropwise to the concentrate until the precipitation was complete. After 3 h of mixture sedimentation, the supernatant was discarded and the precipitates were washed with 20 ml of 0.1 M ammonium hydroxide and then filtered. The residue was the alkaloid, which was dried and weighed. The percentage alkaloid was then calculated as weight of residue x 100/weight of sample taken.

### 2.5 Determination of antioxidant activity

The assay was based on the reduction of Molybdate (VI) to Molybdate (V) by the extract and subsequent formation of green phosphate/MO (V) complex at acid pH as described by Akinmoladun et al. (2007). Ascorbic acid was used as a standard. 1 ml of 0.6 M sulphuric acid, 28 mM of sodium phosphate and 4 mM of ammonium molybdate were added in 20 ml of distilled water and made up to the volume of 50 ml by adding distilled. 0.3 ml of the extract was measured into three separate test tubes. 0.3 ml of the molybdate reagent solution was then added. These tubes were kept incubated at 95 °C for 90 min. After incubation, the tubes were normalized to room temperature for 30 min and the absorbance of the reaction mixture was measured at 695 nm against a blank containing 100 μL of methanol mixed with 900 μL of reagent solution. The antioxidant activity was expressed as mg/g ascorbic acid equivalent (AAE) of dry extract.

### 2.6 Determination of metal chelating capacity

Metal chelating activity was measured by the method described by Chew et al. (2000). 0.1 mM FeSO_4_ and 0.25 mM of ferrozine, forming an Fe^2+^-ferrozine complex, were subsequently added into 0.2 mL of the extract. After incubation at room temperature for 10 min, absorbance of the mixture was recorded at 562 nm. EDTA was used as a positive control.

### 2.7 Statistical analysis

All experiments were performed in triplicate and the outcomes expressed as mean ± standard deviation. The obtained data were subjected to statistical analysis using GraphPad Prism 5.01. A One-way ANOVA was used to compare mean values among the various extracts. P-values less than 0.05 (*P* < 0.05) were considered statistically significant using Tukey's Multiple Comparison Test.

## 3 Results and discussion

### 3.1 Qualitative phytochemical tests

The qualitative phytochemical screening carried out on the aqueous and ethanol extracts of pulp, seeds and whole fruit of *T. tetraptera* showed the presence of flavonoids, alkaloids, tannins, saponins, steroids, terpenoids and phenols in either one or in both extracts as shown in Table 1. The results from the table disclosed the effect of the two solvents (distilled water and ethanol) on the extraction of phytochemicals from *T. tetraptera.* In the two forms of the extracts, greater number of phytochemicals were present in the ethanolic extract than that of the aqueous extract. Thus, 18 phytochemical tests were positive and 3 were negative in the ethanolic extracts whereas 16 phytochemicals tested positive and 5 negatives in the aqueous extracts of the pulp, seeds and whole fruit of *T. tetraptera*. This observation (greater number of phytochemicals revealed in the ethanolic extract than the aqueous extract) could be accounted for by the reason of ethanol having the greater potency to penetrate the cellular membrane and its promotion of rapid physiologic absorption of the extract from the plant material or that the phytochemicals are more soluble in ethanol than that of water. Hence, ethanol extracted more phytochemicals compared to distilled water, making it the suitable solvent for maximum phytochemical extraction from *T. tetraptera*.

**Table 1:** Qualitative phytochemicals tests of *T. tetraptera*.

Phytochemical	Aqueous extract			Ethanolic extract		
	Pulp	Seed	W. fruit	Pulp	Seed	W. fruit
Flavonoids	*+*	*+*	*+*	*+*	*+*	*+*
Alkaloids	*+*	*+*	*+*	*+*	*+*	*+*
Terpenoids	*-*	*-*	*-*	*+*	*-*	*+*
Tannins	*+*	*-*	*+*	*+*	*-*	*+*
Steroids	*+*	*-*	*+*	*+*	*-*	*+*
Saponins	*+*	*+*	*+*	*+*	*+*	*+*
Phenols	*+*	*+*	*+*	*+*	*+*	*+*

**Key**: (+) represents present; (-) represents absence of a particular phytochemical.

*W. fruit = Whole fruit.

### 3.2 Optimization of cold maceration extraction method

This was done by using two different extraction solvents (distilled water and ethanol) to determine the accuracy of the method used and recovery of the analytes (compounds of interest). Owing to the unavailability of certified reference materials for phenols, flavonoids, saponins, alkaloids, antioxidant and metal chelating capacity in the pulp, seeds and whole fruit of *T. tetraptera*, the spike recovery test was a reliable method to test for accuracy. This test was performed by splitting the samples into two portions and a known amount of a standard solution of analyte was added to one portion. The analyte's concentration was determined for both the spiked, *F*, and unspiked portions, *I,* and the percent recovery, %R, was calculated as; %R *= F−I/A* * 100. Where A is the concentration of analyte added to the spiked portion. The use of ethanol as an extraction solvent gave a significantly high recovery ranging between 92% and 108% than that of distilled water (90% and 102%) as shown in Table 2 below. Hence ethanol provided a higher yield than distilled water for all the analytes.

**Table 2:** Recoveries (%) of analytes in aqueous and ethanolic extracts of *T. tetraptera.*

Analyte	Recovery (%)	
	Aqueous extract	Ethanolic extract
Phenols	92.5	94.6
Flavonoids	90	92
Saponins	93	96
Alkaloids	98	102
Antioxidant	96	99.8
Metal chelating capacity	102	108

### 3.3 Quantitative determinations

The quantitative assay was performed for the phytochemicals (phenols, flavonoids, saponins, alkaloids), antioxidant and metal chelating capacity in both the aqueous and ethanolic extracts using UV-Vis Spectrophotometer with standard procedures.

#### 3.3.1 Total phenols, flavonoids, saponins and alkaloids

The concentration of phenols, flavonoids, saponins and alkaloids in both the aqueous and ethanolic extracts of the pulp, seeds and whole fruit of *T. tetraptera* are represented in Tables 3 and 4 respectively. The quantity of phenols, flavonoids, saponins and alkaloids in both extracts were all significantly higher in the pulp than the whole fruit or seeds, suggesting that these bioactive compounds had been largely enriched in the pulp than the other parts of the fruit. Our findings that the aqueous and ethanolic extracts of the pulp contained the highest phytochemical concentrations corroborates the findings by Ekwenye and Okorie (2010), Nwoba (2015) and Uyoh et al. (2013); who estimated the concentration of phenols, flavonoids, saponins and alkaloids in the pulp and seeds of the same plant species. However, this present study has unveiled the concentrations of these major phytochemicals not only in the pulp or seeds but also in the whole fruit (pulp and seeds mixture), with less data in literature.

**Table 3:** Phytochemicals, antioxidant and metal chelating capacity in aqueous extract of *T. tetraptera*.

Sample	Phytochemical				AA (mg AAE/g)	MCC (% EDTA)
	Phenols (mg GAE/g)	Flavonoids (mg QE/g)	Saponins (mg DE/g)	Alkaloids (%w/w)		
Pulp	1.40 ± 0.10^a^	0.67 ± 0.03^a^	3.65 ± 0.03^a^	4.23 ± 0.15^a^	0.41 ± 0.00^a^	4.10 ± 4.76^a^
Seeds	0.15 ± 0.05^b^	0.27 ± 0.03^b^	1.23 ± 0.03^b^	1.30 ± 0.20^b^	0.15 ± 0.01^b^	2.12 ± 4.06^b^
W. fruit	0.94 ± 0.09^c^	0.61 ± 0.03^a^	2.50 ± 0.04^c^	3.93 ± 0.15^a^	0.22 ± 0.02^c^	3.20 ± 2.80^bc^
P-value	0.0293	0.0412	0.0215	0.0315	0.0442	0.0331

*GAE= Gallic acid equivalent, QE= Quercetin equivalent, DE=Diosgenin equivalent, w=Weight. AA= Antioxidant activity, MCC= Metal chelating capacity, AAE= Ascorbic acid equivalent,* W*.* fruit = Whole fruit. Each value is presented as mean ± standard deviation. Means in a column with the same letter superscripts are not significantly different (P > 0.05, Tukey's test), whereas those with different letter superscripts are significantly different (P < 0.05, Tukey's test).

**Table 4:** Phytochemicals, antioxidant and metal chelating capacity in ethanolic extract of *T. tetraptera*.

Sample	Phytochemical				AA (mg AAE/g)	MCC (% EDTA)
	Phenols (mg GAE/g)	Flavonoids (mg QE/g)	Saponins (mg DE/g)	Alkaloids (%w/w)		
Pulp	3.51 ± 0.03^a^	0.87 ± 0.03^a^	4.27 ± 0.03^a^	5.03 ± 0.15^a^	0.50 ± 0.00^a^	5.25 ± 4.76^a^
Seeds	0.70 ± 0.06^b^	0.45 ± 0.03^b^	2.67 ± 0.04^b^	2.37 ± 0.15^b^	0.25 ± 0.01^b^	3.21 ± 4.06^b^
W. fruit	2.95 ± 0.06^c^	0.81 ± 0.03^a^	3.31 ± 0.03^c^	4.77 ± 0.15^a^	0.30 ± 0.02^c^	4.23 ± 2.80^bc^
P-value	0.0224	0.0442	0.0345	0.0391	0.0332	0.0251

*GAE= Gallic acid equivalent, QE= Quercetin equivalent, DE=Diosgenin equivalent, w=Weight. AA= Antioxidant activity, MCC= Metal chelating capacity, AAE= Ascorbic acid equivalent,* W*.* fruit = Whole fruit. Each value is presented as mean ± standard deviation. Means in a column with the same letter superscripts are not significantly different (P > 0.05, Tukey's test), whereas those with different letter superscripts are significantly different (P < 0.05, Tukey's test).

Per gram of each extract, alkaloid seems to be the most abundant bioactive compound having the highest concentration in both the aqueous and ethanolic extracts with flavonoids being the least. Alkaloids have been reported to function as defensive elements against predators, especially mammals because of their general toxicity and deterrence capability as well as analgesic, anti-inflammatory and adaptogenic activities which help to alleviate pains, developed resistance against diseases and endurance against stress (Kaur and Arora, 2015). Flavonoids have also been referred to as nature's biological response modifiers because of the strong experimental evidence of their inherent ability to modify the body's reaction to allergies, virus and carcinogens (Vasantha et al., 2012). Also, Phenols are one of the major groups of non-nutritive dietary components that have been associated with the inhibition of cancer, atherosclerosis, and age-related degenerative brain disorder (Chang et al., 2006). Saponins from plants sources are essential to man owing to their responsibility for some pharmacological effects like anti-inflammatory, antimicrobial, antidiabetic and anticancer, hypocholesterolemia and anticonvulsant in humans (Ali et al., 2011).

#### 3.3.2 Antioxidant activity

The antioxidant activity determination performed on both the aqueous and ethanolic extracts of the pulp, seeds and whole fruit of *T. tetraptera* is displayed in Tables 3 and 4 respectively. In both extracts, the pulp exhibited much antioxidant property as compare to the whole fruit or seeds of *T. tetraptera*. The obtained result is however lower than the 0.94 ± 0.01 mg AAE/g, estimated by Silva et al. (2007), in the ethanolic pulp extract of the same plant species. The difference in antioxidant activity compared to the reported result may be due to the differences in varieties of *T. tetraptera* used (different varieties possess different chemical properties), different growing medium (soil) or the differences in extraction techniques. However, this present study compares the antioxidant property in the pulp, seeds and whole fruit in both the aqueous and ethanolic plant extracts, with limited facts in literature. Antioxidants function as reducing agents, ultimately eliminating free radical intermediates and inhibiting further oxidation (Silva et al., 2007).

#### 3.3.3 Metal chelating capacity

The metal chelating capacity (MCC) was determined using EDTA standard on both the aqueous and ethanolic extracts of *T. tetraptera,* which is presented in Tables 3 and 4 respectively. Based on the data in both Tables 3 and 4, the metal chelating capacity was found higher in the pulp than the whole fruit or seeds, implying that the MCC has been well enriched in the pulp compared to the whole fruit and seeds. Also, the difference in MCC in the pulp, seeds and whole fruit was statistically significant (P < 0.05) for both the aqueous and ethanolic extracts. However, no significant difference in MCC was recorded between the seeds and whole fruit for both extracts. It was evident from the study that, *T. tetraptera* possesses a metal chelating capacity and this might play a protective role against oxidative damaged induced by metal catalyzed decomposition reactions (Joel et al., 2017).

## 4 Conclusion

This study has unveiled that the extract of *T. tetraptera* fruit, which has been reported to be commonly used in the traditional systems of medicine, contains an appreciable amount of phytochemicals and also possesses antioxidant and metal chelating capacities. The phytochemical screening tests revealed the presence of many phytochemicals including phenols, flavonoids, alkaloids, tannins, terpenoids, steroids and saponins in either one or both extracts (aqueous and the ethanolic) of the pulp, seeds and whole fruit of *T. tetraptera*. It was established that the metal chelating capacity was in the order of pulp>whole fruit>seeds*.* It was also discovered from the study that the pulp had significant antioxidant activity and is therefore recommended for antioxidant role than the whole fruit or seeds. The presence of many active phytochemical compounds, antioxidants and metal chelating capacity in the fruit of *T. tetraptera* advocate its antioxidant role and ethno-pharmacological uses in traditional medicine.

## Declarations

### Author contribution statement

Stephen Adusei, Prince Oteng, Richard Q Mensah, Emmanuel Tei-Mensah: Performed the experiments; Analyzed and interpreted the data; Wrote the paper.

John K Otchere: Conceived and designed the experiments.

### Funding statement

This research did not receive any specific grant from funding agencies in the public, commercial, or not-for-profit sectors.

### Competing interest statement

The authors declare no conflict of interest.

### Additional information

No additional information is available for this paper.
